# Can humans smell tastants?

**DOI:** 10.1093/chemse/bjad054

**Published:** 2024-01-04

**Authors:** Shuo Mu, Markus Stieger, Sanne Boesveldt

**Affiliations:** Division of Human Nutrition and Health, Wageningen University and Research, Wageningen, The Netherlands; Division of Human Nutrition and Health, Wageningen University and Research, Wageningen, The Netherlands; Division of Human Nutrition and Health, Wageningen University and Research, Wageningen, The Netherlands

**Keywords:** odor, tastant, fatty acid, ITEX-GC-MS, olfactory discrimination

## Abstract

Although studies have shown that olfaction may contribute to the perception of tastant, literature is scarce or circumstantial, especially in humans. This study aims to (i) explore whether humans can perceive solutions of basic prototypical tastants through orthonasal and retronasal olfaction and (ii) to examine what volatile odor compounds (VOCs) underlie this ability. Solutions of 5 basic tastants (sucrose, sodium chloride, citric acid, monosodium glutamate [MSG], quinine) dissolved in water, and 2 fatty acids (oleic and linoleic acid) dissolved in mineral oil were prepared. Triangle discrimination tests were performed (*n* = 41 in duplicate) to assess whether the tastant solutions can be distinguished from blanks (solvents) through ortho- and retronasal olfaction. Participants were able to distinguish all tastant solutions from blank through orthonasal olfaction. Only sucrose, sodium chloride, oleic acid, and linoleic acid were distinguished from blank by retronasal olfaction. Ethyl dichloroacetate, methylene chloride, and/or acetone were identified in the headspace of sucrose, MSG, and quinine solutions but not in the headspace of water, sodium chloride, and citric acid solutions. Fat oxidation compounds such as alcohols and aldehydes were detected in the headspace of the oleic and linoleic acid solutions but not the mineral oil. We conclude that prototypical tastant solutions can be discriminated from water and fatty acid solutions from mineral oil through orthonasal olfaction. Differences in the volatile headspace composition between blanks and tastant solutions may have facilitated the olfactory discrimination. These findings can have methodological implications for future studies assessing gustatory perception using these prototypical taste compounds.

## 1. Introduction

The flavor is a multifaceted sensory experience that plays a crucial role in the perception and enjoyment of foods and beverages; its perception guides food selection and promotes the ingestion of nutrients (De Graaf and Zandstra 1999; Forde et al. 2013). Flavor encompasses the combination of gustatory, oral-somatosensory, and retronasal olfactory signals (Small 2012). The gustatory system provides information about basic tastes—sweet, sour, bitter, salty, and umami—while ortho- and retronasal smell contribute to the perception of aromas and volatiles. Tastants are molecules that are dissolved in ingested foods and beverages that can bind to taste receptors on the human’s tongue. They are supposed to be non-volatile and thereby non-odorous and to be perceived by the gustatory system only. However, there are animal studies suggesting that certain tastants can be perceived via smell. Bell et al. (1979) found that olfactory bulbectomy in sheep decreased their aversion to sodium salt, while Rhinehart-Doty et al. (1994) demonstrated that rats can discriminate between sucrose solution concentrations by sensory cues other than taste, possibly olfaction. The functional role of the olfactory sense in perceiving taste stimuli has been highlighted in animals, for example, in the conditioned sucrose preference of mice. Zukerman et al. (2009) observed that mice displayed a decreased consumption of sucrose solution after olfactory bulbectomy.

For humans, evidence that olfaction plays a role in the perception of tastant solutions is scarce or circumstantial. Tsuji et al. (2018) showed that taste recognition and detection thresholds increased upon nasal obstruction, and similarly, Mojet et al. (2003) demonstrated reduced salty taste intensity perception upon nasal obstruction. In a recent publication by He et al. (2023), aerosols were observed to be generated during oral processing, which were able to deliver nonvolatile compounds to the nasal cavity, and potentially trigger the olfactory system. In addition, Margulis and colleagues (2023) recently computed that about 14% of bitter molecules are potentially odorous, based on prediction algorithms. Although these studies may suggest that perception of tastant solutions sometimes also encompasses an olfactory component and would thus be a multimodal sensation, these studies do not directly demonstrate that solutions of (basic) tastants have a smell themselves, nor what volatiles would be responsible for that. To our knowledge, only two studies (Mojet et al. 2005; Chen 2013, unpublished data) directly assessed whether humans are able to detect a smell from (basic) tastants, with inconsistent results and small sample sizes. Mojet et al. (2005) reported that participants discriminated between sucrose solutions and water by merely sniffing, and several participants consistently detected 7 out of 10 tastant solutions by olfaction. Similarly, Chen (Chen 2013) observed that participants discriminated monosodium glutamate (MSG) and sucrose solutions from water through orthonasal, but not retronasal olfaction.

Fat taste has been suggested as a sixth basic taste. According to previous studies (e.g. Simons et al. 2011; Galindo et al. 2012; Pepino et al. 2012), there might be specific receptors on the human tongue that respond to fatty acids, though others argue that the sensory experience of fat taste may actually be a combination of sensory modalities including taste, texture and aroma (Stewart et al. 2010; Newman and Keast 2013). Despite these controversies, and unlike the other five basic tastes, there is consistent evidence showing that fatty acids can be smelled. Humans can discriminate fatty acids from mineral oil through ortho- and retronasal olfaction (Chalé-Rush et al. 2007; Bolton and Halpern 2010), can discriminate oleic, linoleic, and stearic acids from each other through retronasal olfaction (Kallas and Halpern 2011), and can describe the smell of these fatty acids (Chukir et al. 2013). Recent reviews provide further overview about the role of olfaction in fat perception (Jaime-Lara et al. 2023; Pirc et al. 2023). To summarize, olfaction may be involved in the detection of solutions of basic tastants and fatty acids in animals and potentially in humans. It is important to assess whether prototypical taste stimuli that are commonly used in psychophysical research (i.e. solutions of sucrose for sweet, NaCl for salt, quinine for bitter, citric acid for sour, and MSG for umami), are perceptible by means of olfaction, as this could have methodological implications for future studies assessing gustatory perception. Dalton et al. (2000) previously examined the potential interaction between gustatory and olfactory modalities and employed saccharin as a pure gustatory stimulus because it lacks volatility and does not offer any olfactory cues. At present, a very limited number of studies explored whether humans can olfactorily detect solutions of prototypical basic tastants and can discriminate between tastant solutions and water based on smell only (Mojet et al. 2005; Chen 2013), and the mechanisms underlying this potential discrimination capability are unclear.

Taste receptors are not only distributed in the oral cavity but also in other regions of the body, such as the gut, large intestine, and the nasal cavity (Behrens and Meyerhof 2006; Finger and Kinnamon 2011). Tastants typically have low volatility and are thus unlikely to be delivered to the nasal cavity. Previous studies hypothesized that olfactory discrimination between basic tastant solutions and water was facilitated by impurities in tastant solutions rather than the tastants themselves (Mojet et al. 2005). Others refuted this hypothesis as they observed that the purity grade of the tastants (e.g. sucrose in reagent grade, non-reagent grade, and food grade) did not influence olfactory discrimination ability, both in mice (Zukerman et al. 2009) and in humans (Chen 2013). However, none of these studies analyzed the headspace of the tastant solutions. Exploring the volatile compounds in the headspace of tastant solutions, which may come from impurities, may help to determine the odor-active compounds that facilitate discrimination between tastant solutions and water and may help to explain the mechanisms underlying the putative ability to detect or discriminate tastants via smell.

This study aims to (i) explore whether humans can perceive solutions of basic prototypical tastants commonly used in psychophysical research through orthonasal and retronasal olfaction, (ii) and if so, to examine what volatile odor compounds (VOCs) underlie this ability. Solutions of five basic tastants (sucrose, sodium chloride, citric acid, monosodium glutamate [MSG], and quinine dissolved in water) and 2 fatty acids (oleic and linoleic acid dissolved in mineral oil) were prepared, and triangle discrimination tests were performed to assess whether the tastant solutions can be distinguished from the blanks (solvents) through ortho- and retronasal olfaction. The headspace composition of the volatile odor compounds was determined using In-Tube Extraction-Gas Chromatography-Mass Spectrometry (ITEX-GC-MS) and linked to the olfactory discrimination ability.

## 2. Materials and methods

### 2.1 Materials

Sucrose, sodium chloride, citric acid, monosodium glutamate (MSG), quinine, oleic acid, linoleic acid, and mineral oil were purchased from Merck KGaA (Darmstadt, Germany). Sucrose, sodium chloride (NaCl), citric acid, MSG, and quinine were stored (as recommended) at room temperature, oleic acid, linoleic acid, and mineral oil were stored (as recommended) at 4 °C before use. Milli-Q water (electrical resistivity 18.2 MΩ·cm at 25°C) produced using the Arium 611UF ultrapure water system (Sartorius Stedim Biotech GmbH, Göttingen, Germany) was used as a solvent for five tastants and mineral oil was used as a solvent for fatty acids. The concentrations of tastant solutions were chosen according to previous studies and their occurrence in foods and beverages (Chukir et al. 2013; Mojet et al. 2005). High concentrations of tastants and fatty acids were chosen that are easily perceivable by humans through taste, while still remaining within an ecological relevant tastant concentration range, so concentrations that are high but occur in common foods and beverages. The purity of solutes and final concentration of solutions are shown in Table 1.

**Table 1.** The purity of solutes, solvents, and concentration of tastant and fatty acid solutions.

Solutes	Solvents	Purity of solutes	Concentration (g/100 g)
Sucrose	Milli-Q water	≥99.5%	25
Sodium chloride	Milli-Q water	≥99.0%	3
Citric acid	Milli-Q water	≥99.0%	5
MSG	Milli-Q water	≥98.0%	1
Quinine	Milli-Q water	≥90.0%	0.0083
Oleic acid	Mineral oil (neat)	≥90.0%	40
Linoleic acid	Mineral oil (neat)	58.0–74.0%	40

Brown glass bottles (150 mL) were used for orthonasal testing, and specially designed cups (150 mL) were used for retronasal testing (Pirc et al. 2022). The setup and usage of special designed cups are shown in Supplementary Fig. S1. Each brown bottle contained 60 g of tastant solutions or water or 50 g of fatty acid solutions or mineral oil, and each special designed cup contained 80 g of water solutions or 60 g of oil solutions. The amounts of tastant and fatty acid solutions were calculated based on their density to ensure a consistent volume in the bottles or cups. The solutions were prepared one day before testing and stored at 4 °C. All samples were taken out of the refrigerator 1 h before testing to come to room temperature.

### 2.2 Sensory experiments

#### 2.2.1 Participants

Forty-two participants (mean age 24.0 ± 6.9 years; 8 males; mean BMI 21.6 ± 2.8 kg/m^2^) took part in the study. All participants were non-smokers, not pregnant, not breastfeeding, nor currently on a calorie-restricted diet or have been in the past 2 months. All of them had a normal olfactory function according to the 16-item odor identification part of the Sniffing’ Sticks test (score of ≥12; Hummel et al. 2007). All participants finished the orthonasal olfactory tests. One participant quit during the retronasal olfactory test session so that 41 participants finished the retronasal olfactory tests. Participants were asked not to eat or drink anything other than water 1 h prior to testing, nor wear any scented products on the day of testing. Their demographic information (age, gender, height, and weight) was collected through an online questionnaire. Written informed consents were provided by all participants prior to participation, financial reimbursements were transferred to participants when they completed all sessions. The study was exempt from review by the Medical Research Ethical Committee (number 2022-118-SBSEB-prc) according to the “Medical Research Involving Human Subjects Act” of The Netherlands (WMO in Dutch). The study was conducted in agreement with the ethics regulations laid out in the Declaration of Helsinki (originally adopted in 1964 and amended in Fortaleza in 2013).

#### 2.2.2 Study procedure

Sensory assessments were conducted in individual sensory booths at Wageningen University, the Netherlands. The sensory booths are well-ventilated to ensure an odor-free environment. Participants attended 4 sessions of 30–40 min. The first session contained the Sniffing’ Sticks test, and a training on how to use the special designed cups for retronasal olfactory testing (see Supplementary Fig. S1). The second and third sessions consisted of retronasal olfactory triangle discrimination tests. In each session, 7 sets of triangle comparisons were performed to compare each of the solutes (Table 1) to its solvent. The data obtained from the 2 sessions were treated as duplicate measures while different sample comparisons were performed (e.g. when triangle comparison ABB was performed in the second session, AAB performed in the third session was considered as its duplicate. Presentation order within triangles (i.e. ABB, BAB, BBA) were randomized over participants in each session. The fourth session contained 14 sets of triangle comparisons (7 sets in duplicate) for orthonasal olfaction test in random order. An example of the study design for all sessions is shown in Supplementary Table S1.

The olfactory triangle discriminations were between tastant (or fatty acids) solution and blank (solvent, respectively). In the test, participants were instructed to smell all 3 samples in the order samples were presented, and then select the different (odd) one out. Subsequently, participants had to answer the following questions: “Did you distinguish the samples based on intensity of the odor, quality of odor, or did you just guess?” If participants answered “intensity” they were then asked “Did you perceive the odd sample as more, or less intense compared to the other samples?.” Finally, they were asked, “Which taste do you associate with the sample you smelled?,” with response options sweet, salty, bitter, sour, umami, sour, fat, other, or nothing. An inter-trial interval of approximately 1 minute was used between each triangle test. Participants were encouraged to smell their own skin between trials to prevent adaptation during the intervals.

### 2.3 Characterization of volatile compound composition

The characterizations of volatile compound composition were performed for all samples used in the sensory test. The headspace volatile compound composition was determined by ITEX-GC-MS (In-Tube Extraction Gas Chromatography–Mass Spectrometry). 5 mL liquid sample was injected into a 20-mL vial. Vials were sealed and stored at 4 °C overnight and were removed from the refrigerator one hour before analysis. An auto-sampler (TriPlus, Thermo, USA) was employed for automatically loading and extracting samples. The vial was incubated at 60 °C for 10 min before analyzing. The headspace of samples was extracted using a Tenax tube (GR 80/100, Buchem B.V., Minden, The Netherlands.) Extraction was set as 10 times with 1.3 mL each time. The injection was set as 1 mL headspace coupled with the desorption from the Tenax tube.

A gas chromatograph system (Trace 1300, Thermo, USA) coupled with a single quadrupole mass spectrometer (ISQ 7000, Thermo, USA) was employed to analyze the volatile composition of the headspace. Rxi-5SIL MS column (30 m × 0.25 mm, df = 1.0 µm, Restek GmbH, Schaberweg, Germany) was used in the analysis. The carrier gas was hydrogen, at 2.17 mL/min. The initial oven temperature was 40 °C and was maintained for 2 min. The temperature was then increased to 250 °C at 30 °C/min and held for 2 min. The mass spectrometry detection setting was a full scan model, with a mass range of 25–250 *m/z*. All samples were measured in triplicate. Total ion chromatograms (TICs) were recorded and used for further analysis.

The chromatograms were recorded and analyzed using Thermo Scientific Dionex Chromeleon® 7.2 chromatography data system software. Volatile compounds were identified by comparing their mass spectra and retention indices with the National Institute of Standards and Technology database. Measurements were performed in triplicate for fatty acid solutions and mineral oil, and the compounds detected in all replicates were recorded. As for tastant solutions and Milli Q water, only a few compounds were detected in the headspace based on our preliminary tests. To ensure measurement accuracy, 2 batches of samples were prepared, and each sample was measured in triplicate (yielding 6 measurements per sample). The compounds detected in at least five measurements were recorded. The mean values of the total ion currents (TICs) were calculated and used for further data analysis.

### 2.4 Statistical data analysis

Corresponding triplets of the triangle discrimination tests (e.g. AAB and ABB) were tested for independence according to Smith’s test (1981). For none of them, the difference in proportions of correct responses between the two replications was significant, and the replicates could thus be pooled, resulting in *n* = 84 observation for the retronasal test and *n* = 82 observations for the orthonasal test. The number of correct responses was summed up, and the significance level (*P*) was calculated according to binominal tests. According to answers from the additional question of triangle discrimination tests, the proportion of responses to question 2 (discrimination based on odor intensity, quality, or guess) and question 3 (odor associate with sweet, salty, bitter, sour, umami, sour, fat, other, or nothing) were calculated based on the correct responses in triangle discrimination test. One-way ANOVA followed by the Duncan test was performed to analyze differences in TIC of volatile compounds between samples using IBM SPSS Statistics 25.0 (SPSS Inc., Chicago, IL). A significance level of *P* < 0.05 was chosen for all analyses.

## 3. Results

### 3.1 Olfactory discrimination ability of tastant and fatty acids solutions

**Fig. 1.**
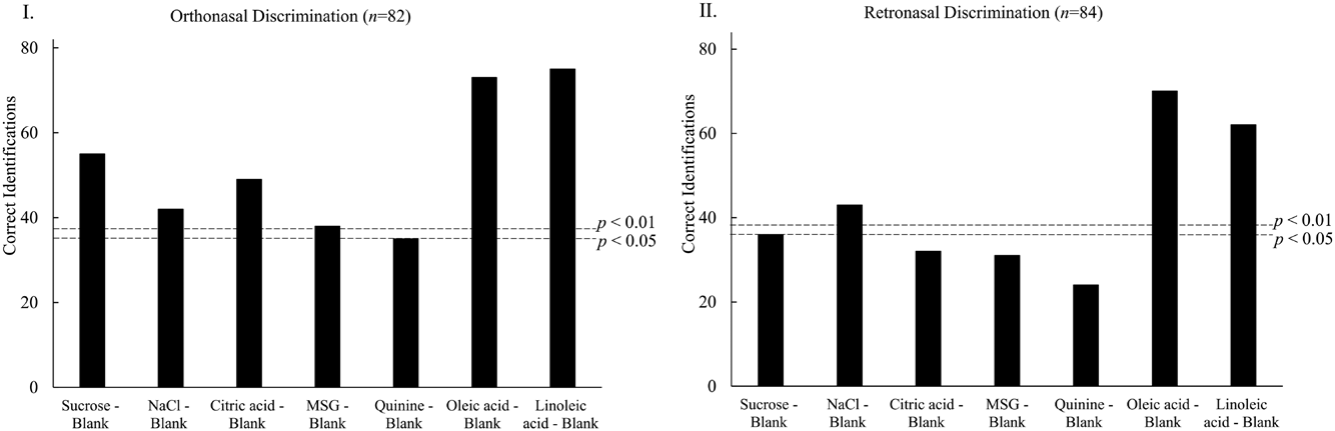
Number of correct identifications for triangle discrimination tests. (I): Orthonasal olfactory discrimination for prepared solutions and blanks (*n* = 82, 41 participants in duplicate). (II): Retronasal olfactory discrimination for prepared solutions and blanks (*n* = 84, 42 participants in duplicate). Dotted lines indicate the minimum number of correct identifications required at different significance levels. Milli Q water was used as blank for tastant solutions and mineral oil was used as blank for fatty acid solutions.

Fig. 1 depicts the results of olfactory triangle tests conducted for orthonasal discrimination (I) and retronasal discrimination (II). Participants were able to discriminate between tastant or fatty acid solutions and the blank (water or mineral oil) through orthonasal olfaction (all *P-*values < 0.01, except for the comparison between quinine and the blank, *P* = 0.048). By means of retronasal olfaction, participants discriminated sucrose (*P* = 0.043), NaCl (*P* < 0.01), oleic acid (*P* < 0.01), and linoleic acid solutions (*P* < 0.01) from blank, but they were unable to discriminate citric acid (*P* = 0.213), MSG (*P* = 0.286), and quinine solutions (*P* = 0.850) from water.

**Fig. 2.**
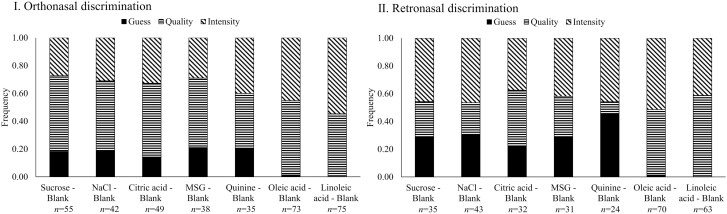
Reasons that participants provided for olfactory discrimination between solutions and blank. (I): Orthonasal discrimination. (II): Retronasal discrimination. All results were calculated based on correctly discriminated trials only; n indicates the number of correct responses for each sample comparison; Milli Q water was used as blank for tastant solutions and mineral oil was used as blank for fatty acid solutions.

The proportions of responses (based on correct responses only) indicating whether participants based their judgment on odor intensity, quality, or guess are presented in Fig. 2. Regarding orthonasal discrimination, more participants attributed their discrimination ability to odor quality (50–55%) as opposed to odor intensity (27–33%) for all comparisons except for quinine, oleic acid, and linoleic acid trials, where odor intensity (40–55%) and odor quality (40–53%) contributed equally to the discrimination. In terms of retronasal discrimination, more participants indicated that odor intensity (42–47%) contributed to the discrimination of sucrose, NaCl, MSG, and quinine, whereas both odor quality and odor intensity contributed equally to the discrimination of citric acid and oleic acid. Furthermore, a higher proportion of participants made guesses in retronasal tests (22–46%) for tastant solutions compared to the corresponding orthonasal tests (14–21%).

**Fig. 3.**
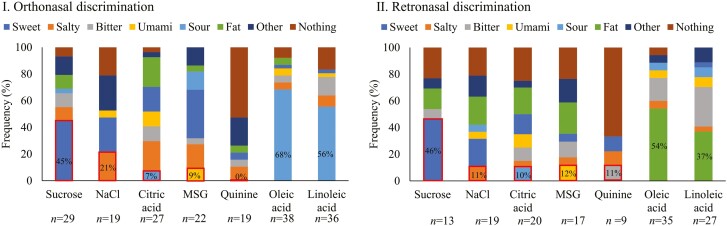
Frequency of the association of the odor of the tastant solution with the taste quality of the tastant. (I): Orthonasal discrimination. (II): Retronasal discrimination. Only correctly discriminated trials were included. The red boxes highlight the frequency when the odor of the tastant solutions matches the taste quality of the tastant. For colour figure refer to the online version.

In orthonasal and retronasal trials, respectively, 45% and 46% of participants associated the odor of the sucrose solution with sweetness trial (Fig. 3). For the other 4 tastants, only very few participants associated the odor of the tastant solution with the taste quality of the tastant trial (21% and 11% for NaCl, 7% and 10% for citric acid, 9% and 12% for MSG, 0% and 11% for quinine, in orthonasal and retronasal trials, respectively). For fatty acids, many participants associated the odor of oleic (68%) and linoleic acid solutions (56%) with a sour taste in the orthonasal condition, whereas they associated the odor of oleic (54%) and linoleic acid (37%) solutions with fat taste in the retronasal condition.

### 3.2 Headspace volatile compound composition of tastant and fatty acid solutions

The compositions of volatile compounds in the headspace of tastant solutions and Milli Q water are presented in Table 2. Trichloromethane and diethyl azodicarboxylate were identified in the headspace of all five tastant solutions and Milli Q water. Trichloromethane was found to be significantly (*P* < 0.05) more abundant in the headspace of sucrose and quinine solutions compared to Milli Q water. No significant differences (*P* > 0.05) were observed in the abundance of diethyl azodicarboxylate between tastant solutions and Milli Q water. Several compounds were identified only in the headspace of tastant solutions when compared to Milli Q water. Acetone and ethyl dichloroacetate were identified only in the headspace of the sucrose solution, while ethyl dichloroacetate and methylene chloride were detected only in the headspace of the quinine solution. Acetone was identified only in the headspace of the MSG solution. No compounds were identified in the headspace of the NaCl and citric acid solutions similar to Milli Q water.

**Table 2.** Peak area (total ion chromatogram, ×10^6^) of compounds detected in headspace of tastant solutions and Milli Q water.

Compound	CAS Number	Sucrose	NaCl	Citric acid	MSG	Quinine	Milli Q Water	Odor quality
Trichloromethane	67-66-3	254.8 ± 29.9*	37.3 ± 12.2	36.1 ± 8.9	49.1 ± 9.0	398.5 ± 53.3*	64.5 ± 17.2	hay
Diethyl azodicarboxylate	1972-28-7	47.4 ± 6.5	47.7 ± 11.6	47.2 ± 9.9	45.5 ± 8.9	43.4 ± 13.6	53.1 ± 8.3	*N*
Acetone	67-64-1	21.8 ± 4.6	-	-	64.3 ± 4.9	-	-	chemical, ether, hay, nauseating, pungent, wood
Ethyl dichloroacetate	535-15-9	0.9 ± 0.5	-	-	-	4.5 ± 1.9	-	*N*
Methylene chloride	75-09-2	-	-	-	-	3.1 ± 0.8	-	*N*

Results are expressed as mean ± SD (*n* = 5 or 6). Compounds that were detected in ≥5 measurements are reported. *N*: Not known. * Denotes significant differences in peak area of volatile compounds between a tastant solution and Milli Q water (independent samples *T* test, *P* < 0.05).

The compositions of volatile compounds in the headspace of fatty acid solutions and mineral oil are presented in Table 3. The number of compounds detected in the headspace of oleic acid solution (26) was comparable to linoleic acid solution (27), and both were higher compared to mineral oil (15), demonstrating that fatty acid solutions had more abundant headspace volatile composition. Acids, alcohols, aldehydes, esters, and ketones were identified in all samples. *Trans*-2-octen-1-ol, 2-penten-1-ol, 3-octen-2-ol, isoamyl acetate, methyl anisole, 3-methyl-butanal, ethyl 2-methylbutyrate, 2-butanone, 3-methyl-thiophene, butanoic acid, methyl ester, 1-butanol, 2,3-pentanedione, acetaldehyde, 2-methyl-butanoic acid, and hexanal were identified in the headspace of both fatty acid solutions but were absent in the headspace of mineral oil. When comparing the headspace composition of oleic acid solution with mineral oil, 1-octen-3-one, acetone, 2-butenal, decanoic acid, ethyl ester, 2-heptanone, 1-butanol were more abundant, and 2-methyl-2-butenal were less abundant in the headspace of oleic acid solutions. When comparing the headspace composition of linoleic acid with mineral oil, acetone, diacetyl, 2-hexanone, 2-pentanone, 1-propanol, ethyl 2-methylbutyrate, 2-butanone, 3-methyl-thiophene, and acetaldehyde were more abundant and 2-methyl-2-butenal was less abundant in the headspace of linoleic acid solution.

**Table 3.** Peak area (total ion chromatogram, ×10^6^) of compounds detected in the headspace of fatty acid solutions and mineral oil.

Compound Name	CAS Number	Oleic acid	Linoleic acid	Mineral oil	Odor quality
***Acids***					
Butyric acid	107-92-6	0.5 ± 0.1	0.9 ± 0.2	0.3 ± 0.1	butter, cheese, must, rancid, sour, sweat
2-Methylbutanoic acid	116-53-0	-	0.4 ± 0.1	-	butter, cheese, fermented, rancid, sour, sweat
***Alcohols***					
Ethanol	64-17-5	8.8 ± 1.5	6.9 ± 4.3	14.7 ± 3.1	alcohol, floral, ripe apple, sweet
1-Propanol	71-23-8	9.9 ± 0.4*	11.2 ± 7.6*	1.7 ± 1.3	alcohol, candy, must, plastic, pungent, ripe fruit, rum, sweet
*trans*-2-Octen-1-ol	18409-17-1	1.0 ± 0.2	0.3 ± 0.1	-	medicine, oil, plastic, soap
2-Penten-1-ol	20273-24-9	0.6 ± 0.1	0.5 ± 0.1	-	grass
3-Octen-2-ol	76649-14-4	0.2 ± 0.1	1.1 ± 0.1	-	N
1-Butanol	71-36-3	0.7 ± 0.1	-	-	alcohol, fermented, fruit, medicine, phenol, putrid, solvent, sweat
***Aldehydes***					
2-Butenal	4170-30-3	2.9 ± 1.1*	2.8 ± 1.2	0.5 ± 0.2	pungent
2,6-Nonadienal	557-48-2	0.3 ± 0.1	0.2 ± 0.1	0.2 ± 0.1	cucumber, green, lettuce, melon, wax
2-Methyl-2-butenal	1115-11-3	0.8 ± 0.4*	-	1.1 ± 0.1	apple, fruit, grass, green, solvent
3-Methyl-butanal	590-86-3	1.5 ± 0.5	1.5 ± 0.2	-	acrid, almond, chocolate, cocoa, corn flakes, fermented, malt, pungent, sweat, sweet
Acetaldehyde	75-07-0	-	0.7 ± 0.1	-	ether, floral, fruit, green apple, pungent, sweet
Hexanal	66-25-1	-	2.3 ± 0.4	-	apple, cut grass, fresh, fruit, grass, green, oil
***Esters***					
Decanoic acid, ethyl ester	110-38-3	0.4 ± 0.1*	0.2 ± 0.0	0.2 ± 0.0	brandy, burnt, fruit, geranium, grape, nut, pear, pleasant, soap
Isoamyl acetate	123-92-2	0.7 ± 0.1	0.9 ± 0.0	-	apple, banana, fruit, glue, pear, sweet, yeast
Ethyl 2-methylbutyrate	7452-79-1	0.2 ± 0.1	1.3 ± 0.1	-	anise, apple, bubble gum, fruit, kiwi, strawberry
Butanoic acid, methyl ester	623-42-7	1.3 ± 0.1	-	-	apple, banana, cheese, ester, floral
***Ketones***					
Acetone	67-64-1	58.5 ± 1.1*	23.5 ± 12.3	23.4 ± 6.2	chemical, ether, hay, nauseating, pungent, wood
Diacetyl	431-03-8	6.1 ± 0.1*	10.2 ± 1.8*	1.1 ± 0.1	butter, caramel, cheese, cream, fruit, strawberry, sweet, yogurt
1-Octen-3-one	4312-99-6	0.9 ± 0.8*	0.2 ± 0.1	0.1 ± 0.0	boiled mushroom, earth, green, metal, mushroom, sharp
2-Heptanone	110-43-0	4.0 ± 0.5*	1.9 ± 0.2	0.5 ± 0.0	bell pepper, blue cheese, cinnamon, fruit, green, nut, sweet
2-Hexanone	591-78-6	51.3 ± 0.3*	81.6 ± 9.1*	1.4 ± 0.3	ether
2-Pentanone	107-87-9	0.7 ± 0.0	20.4 ± 1.0*	0.9 ± 0.2	burnt plastic, ether, fruit, kerosine, orange peel, pungent
2-Butanone	78-93-3	0.2 ± 0.0	2.1 ± 1.3	-	butterscotch, ether, fragant, fruit, pleasant, solvent, sweet
2,3-Pentanedione	600-14-6	-	1.1 ± 0.2	-	bitter, butter, caramel, cream, fruit, strawberry, sweet, wine
***Others***					
2-Furfuryl-furan	1197-40-6	0.3 ± 0.0	0.5 ± 0.1	0.4 ± 0.1	N
Dimethyl-Sulfide	75-18-3	18.8 ± 7.7	22.2 ± 12.4	24.2 ± 6.3	cabbage, gasoline, organic, sulfur, wet earth
Methyl anisole	10568-38-4	0.4 ± 0.1	1.1 ± 0.3	-	N
3-Methyl-thiophene	616-44-4	0.8 ± 0.0	1.6 ± 0.0	-	astringent, burnt, plastic

Results are expressed as mean ± SD (*n* = 3). Compounds detected in all 3 measurements are reported. *N*: Not known. * Denotes significant differences in peak area between a fatty acid solution and minerals oil (independent samples *T* test, *P* < 0.05).

## 4. Discussion

This study aimed to (i) explore whether humans can perceive solutions of basic prototypical tastants commonly used in psychophysical research through orthonasal and retronasal olfaction, (b) and, if so, to examine what volatile odor compounds (VOCs) underlie this ability.

### 4.1 Humans can discriminate between tastants and fatty acid solutions from blanks through olfaction

The study demonstrated that participants distinguished all prototypical tastant solutions from blank (water) through orthonasal olfaction. Only sucrose, sodium chloride, oleic acid, and linoleic acid were distinguished from blank by retronasal olfaction. Previous studies investigated the olfactory discrimination ability of humans between tastant solutions at suprathreshold and blanks. Mojet et al. (2005) investigated the orthonasal detection of tastant solutions and reported that “considerable number of subjects (21 out of 41) could regularly detect 7 of the 10 tastants by olfaction.” However, they reported their result as percentages of odor detection after correction for chance guessing without describing how the correction was performed. In an unpublished study, Chen (2013) explored the orthonasal and retronasal perception of tastant solutions by performing ortho- and retronasal olfactory triangle discrimination test between tastant solutions and water. Both studies indicated that only sucrose and MSG solutions were distinguishable from the blanks by orthonasal olfaction. Consistent with these findings, our study confirmed the discriminability of sucrose and MSG solutions at suprathreshold levels from water, orthonasally and retronasally. Furthermore, our study revealed that NaCl, citric acid, and quinine solutions could be discriminated from water through orthonasal olfaction (Fig. 1-I), which is in contrast to the studies of Chen (2013) and Mojet et al. (2005). Moreover, Chen reported that none of the five basic tastant solutions were discriminated from water through retronasal olfaction, while our results show that sucrose and NaCl solutions could be discriminated from water through retronasal olfaction. The disparity in findings may be attributed to differences in experimental design and data analysis. Chen (2013) used 30 participants to perform the discrimination tests without replicates and without specifying the sample comparison design (AAB or ABB). Mojet et al. (2005) recruited 41 participants and performed 4 alternative forced-choice tests without replicates. Our study recruited 41 (orthonasal) or 42 (retronasal) participants and performed measurements in duplicate (AAB and ABB are considered duplicates for one sample comparison in our study design), which resulted in 82 or 84 observations for each sample comparison. Furthermore, Chen (2013) compared discrimination response accuracy (%) with chance level (33% for the triangle test) through one-sample *t* tests. We summed up the number of correct responses for each sample comparison and calculated the significance level (*P*) according to binominal tests as commonly done for triangle tests. Another factor that may influence the olfactory perception of tastant solution is the purity of blank water. Although Mojet et al. (2005) indicated that the purity of water (such as demineralized, double-distilled, and Evian water) had no significant impact on the discriminability of most tastants at both individual and group levels, they observed that the use of double-distilled water enhanced the orthonasal discrimination ability of MSG solution, while the use of Evian water diminished it. Our study used Milli Q water, which is ultrapure water. The different waters used in these studies may have influenced the olfactory perception of tastant solutions; however, more chemical analyzes are necessary to further verify the difference in headspace volatile compound composition of water differing in purities as well as their olfactory perception.

Regarding fatty acids, our results (Fig. 1) are consistent with previous findings that oleic and linoleic acid can be distinguished from blank mineral oil through both orthonasal and retronasal olfaction (Chalé-Rush et al. 2007; Bolton et al. 2010). Furthermore, our findings differ slightly from Bolton and Halpern (2010), who observed that in the discrimination of oleic acid solution, more correct responses were obtained through orthonasal discrimination compared to retronasal discrimination. In our study, we observed this phenomenon for both oleic acid and linoleic acid. This difference could be attributed to the fact that retronasal olfactory thresholds for both oleic and linoleic acids were higher than orthonasal olfactory thresholds (Chalé-Rush et al. 2007), making the perception of the odor of fatty acids easier through orthonasal olfaction compared to retronasal olfaction.

Participants indicated that both odor intensity and odor quality contributed to olfactory discrimination. A higher number of participants guessed during retronasal discrimination tests compared to orthonasal discrimination tests for the tastant solutions (Fig. 2). This could be attributed to the in general lower sensitivity of retronasal olfaction compared to orthonasal olfaction (Heilmann and Hummel 2004). A previous study (Pirc et al. 2022) reported a lower odor intensity of milk through retronasal olfaction compared with orthonasal perception. It is possible that the odor of the tastant solutions in the current study was not strong enough to be reliably detected through retronasal olfaction, making retronasal discriminations more challenging. These findings align with our discrimination results (Fig. 1), where we observed more correct responses in orthonasal discrimination compared to relative retronasal discriminations. It should be noted, however, that our study design was not suited for a direct comparison between ortho- and retronasal results, as we utilized different containers for the 2 pathways (smelling from a glass bottle in the orthonasal comparisons and inhaling via a straw from a specially designed cup in the retronasal conditions).

To summarize, our study suggests that humans are capable of discriminating the headspace of tastant or fatty acid solutions from blank through orthonasal olfaction. These findings might have methodological implications for future studies. The prototypical taste compounds (sucrose, sodium chloride, citric acid, monosodium glutamate [MSG], quinine) used in our study apparently have an odor that is different from water. This suggests that future studies aimed at the exploration of solely gustatory perception in humans should restrict olfactory perception by nasal blockage.

### 4.2 The perceived odor of tastant solutions is not associated with their taste quality

For all tastant solutions, olfactory discrimination was not associated with the specific taste quality of the tastant solution as less than 20% of participants associated the odor of the tastant solution correctly with the taste quality of the tastant solution, with the exception of sucrose solutions, where almost half of participants indicated that they perceived a sweet smell of sucrose solution. Previous animal studies have shown that mice are capable of sensing the odor of sucrose solutions. Rats can discriminate among sucrose solution concentrations by cues other than taste, possibly by olfaction (Rhinehart-Doty et al. 1994). Interestingly, the preference for sucrose solution still remained even when sweet taste receptor T1R3 was genetically knocked out, and such preference decreased when olfaction was blocked (Zukerman et al. 2009). These findings suggest that sucrose solutions emit odors, and those may influence the nutritional behavior of mice.

Regarding fatty acids, our study found that many participants associated the odor of oleic (68%) and linoleic acid (56%) solutions with sourness in the orthonasal condition, whereas they associated them with fat taste (54% and 37% for oleic and linoleic acid, respectively) in the retronasal condition. This seems in line with the Volatile Compounds in Food Online database (https://www.vcf-online.nl), where oleic and linoleic acids have been reported to smell fatty and rancid, which describes the retronasal odor of oleic and linoleic acids as oily, olive oil, sunflower (Chukir et al. 2013). In contrast to retronasal olfactory perception, the orthonasal odors of oleic and linoleic acids were more often associated with sourness. It is known that the same volatile molecules can be perceived differently at various concentrations (Gross-Isseroff and Lancet 1988) and between ortho- versus retronasal routes. Furthermore, the disparity in perceived odor quality between orthonasal and retronasal olfaction may be influenced by anatomical differences (Zhao et al. 2004) and variations in the adsorption environment (Heilmann et al. 2004) along these two routes.

In conclusion, although our study shows that humans are able to distinguish between tastant solutions and blank by means of smell, they are not able to associate the odor of the tastant solution to its associated taste quality, except for fatty acid solutions.

### 4.3. Differences in volatile compound composition between headspaces of tastant and fatty acid solutions and blank might contribute to odor discrimination ability

Our study profiled volatile compound composition in the headspace of tastant solutions and Milli Q water to explore what volatile compounds contribute to olfactory discrimination. Acetone, which presents odor qualities such as ether, hay, and pungent, was identified only in the headspace of sucrose and MSG solutions. The presence of acetone might be related to the manufacturing process of sucrose as acetone might have been used as a (co-) solvent during sucrose purification (US4116712A - Solvent refining of sugar). The residual acetone may have contributed to odor discrimination between sucrose and MSG tastant solutions and Milli Q water. We observed that chlorinated compounds were present in all samples. Specifically, trichloromethane was identified in Milli Q water and all tastant solutions. Peak areas of trichloromethane were significantly higher in the headspace of sucrose and quinine solutions compared to water, whereas ethyl dichloroacetate and methylene chloride were only identified in the headspace of quinine solutions. Chlorinated compounds were previously identified in drinking (Furlong and D’Itri 1986) and public water supplies (Aguilera-Herrador et al. 2008), their presence in water originates from water disinfection treatments (Trussell and Umphres 1978; Golfinopoulos 2000). We do not have an explanation for the observed differences in the relative abundance of chlorinated compounds between tastant solution and water, but these differences might have contributed to olfactory discrimination. Diethyl azodicarboxylate was identified in all tastant solutions and water with similar abundances, so its presence probably did not influence olfactory discrimination. We acknowledged that NaCl and citric acid solutions were discriminated from water through olfaction, but we did not find any differences in volatile composition between their headspaces. We cannot link olfactory discrimination to headspace composition for these two (out of 5) tastant solutions. The headspace analysis method (ITEX-GC-MS) used might not have been sufficiently sensitive to extract and detect all volatile compounds present in the headspace. More studies with different analysis and extraction methods are needed to further verify potential differences in volatile composition between the headspace of tastant solutions and water.

Previous studies investigated the ortho- and retronasal olfactory perception of fatty acids and concluded that both oleic and linoleic acid have distinguishable odors compared to blank mineral oil (Chalé-Rush et al. 2007; Bolton and Halpern 2010). However, these studies did not specify the volatile compound composition in the headspace. In our study, we confirmed that humans can discriminate the odor of oleic and linoleic acid from mineral oil using both ortho- and retronasal olfaction and extended this to identify the volatile compounds that may facilitate this discrimination ability. Several alcohols and aldehydes, including trans-2-octen-1-ol, 2-penten-1-ol, 3-octen-2-ol, 1-butanol, 3-methyl-butanal, acetaldehyde, and hexanal, were only identified in oleic and linoleic fatty acid solutions but not the blank. Furthermore, 1-propanol, diacetyl, and 2-hexanone were identified with significantly larger peak areas in fatty acid solutions compared to mineral oil. These compounds were found to be odor-active (Table 3), which likely facilitated olfactory discrimination between the fatty acid solutions and the mineral oil. The alcohols and aldehydes are well-known oxidation products of fatty acids (Frankel et al. 1981; Kanazawa et al. 1983). Cao et al. (2014) suggested that several aldehydes, such as octanal, nonanal, decanal, and 2-decenal, could serve as oxidation indicators for oleic acid, while hexanal was closely associated with the oxidation of linoleic acid. We suggest that oxidation products of fatty acids contributed to the olfactory discrimination between fatty acid solutions and mineral oil.

We acknowledge that our study focused on qualitative rather than quantitative analysis of the volatile compound composition in the headspace of tastant and fatty acid solutions. It is important to note that volatile compounds contribute to odor perception only when their concentration exceeds the detection threshold. We can only speculate on whether the identified volatile compounds actually influenced olfactory perception, as concentrations of volatile compounds were not quantified. We were unable to obtain odor activity values since the area under the curve rather than the absolute concentration of compounds was determined. Future studies should determine odor activity values to validate our current findings.

## 5. Conclusions

Our study demonstrates that humans can discriminate between solutions of all 5 basic tastants and fatty acids from blanks through orthonasal olfaction and can distinguish solutions of sucrose, NaCl, and 2 fatty acids from blank. The perceived odor qualities of tastant solutions are not associated with their taste quality, whereas the perceived odor qualities of fatty acids are associated with fat. Differences in volatile compound composition between headspaces of solutions and blank might have facilitated olfactory discrimination between tastant and fatty acid solutions and blanks. These findings may have methodological implications for future studies assessing gustatory perception and warrant further investigations to explore how olfaction contributes to taste and fat perception.

## Supplementary Material

bjad054_suppl_Supplementary_Material

## Data Availability

The data underlying this article will be shared on reasonable request to the corresponding author.
