# Supplementary material for: Can humans smell tastants?
Source: Chem Senses. 2024 Jan 4;49:bjad054. doi: 10.1093/chemse/bjad054 (PMC10807988; doi:10.1093/chemse/bjad054)
Supplement: bjad054_suppl_Supplementary_Material [file bjad054_suppl_supplementary_material.docx]

**Figure S1.** The specially designed cups for retronasal olfactory testing. It contains a plastic cup, a silica gel lid with two holes, and a plastic straw plugged in one of the holes. The straw is positioned just above the liquid surface. When using the cup, participant first block their nose with a nose clip, then insert the straw in their mouth, inhale the air through the straw using their mouth, then remove the nose clip, and finally exhale via their nose.


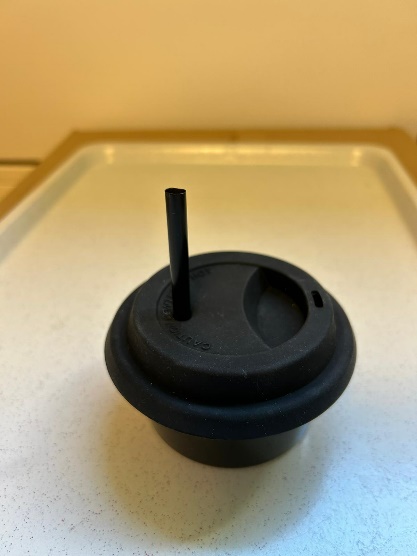


**Table S1**. One example of detailed sample comparisons for each session. The italics in session 3 are considered as duplicate comparisons from session 2 for retronasal test. The italics in session 4 are considered as duplicate comparisons for orthonasal test.

| Session 1  Screening and training. | Sniffin’ Sticks test | | |
| --- | --- | --- | --- |
|  | Training on specially designed cups for retronasal olfactory testing | | |
|  | Pilot test | | |
| Session 2  Retronasal triangle discrimination test | Sample comparison design | | |
|  | Sucrose | Sucrose | Milli Q water |
|  | Sodium chloride | Milli Q water | Sodium chloride |
|  | Milli Q water | Citric acid | Citric acid |
|  | MSG | Milli Q water | MSG |
|  | Quinine | Quinine | Milli Q water |
|  | Mineral oil | Oleic acid | Oleic acid |
|  | Linoleic acid | Linoleic acid | Mineral oil |
| Session 3  Retronasal triangle discrimination test | Sample comparison design | | |
|  | *Milli Q water* | *Milli Q water* | *Sucrose* |
|  | *Milli Q water* | *Milli Q water* | *Sodium chloride* |
|  | *Citric acid* | *Milli Q water* | *Milli Q water* |
|  | *Milli Q water* | *MSG* | *Milli Q water* |
|  | *Milli Q water* | *Milli Q water* | *Quinine* |
|  | *Oleic acid* | *Mineral oil* | *Mineral oil* |
|  | *Mineral oil* | *Linoleic acid* | *Mineral oil* |
| Session 4  Orthonasal triangle discrimination test | Sample comparison design | | |
|  | Sucrose | Milli Q water | Sucrose |
|  | Sodium chloride | Sodium chloride | Milli Q water |
|  | Citric acid | Milli Q water | Citric acid |
|  | Milli Q water | MSG | MSG |
|  | Quinine | Quinine | Milli Q water |
|  | Oleic acid | Mineral oil | Oleic acid |
|  | Linoleic acid | Linoleic acid | Mineral oil |
|  | *Milli Q water* | *Milli Q water* | *Sucrose* |
|  | *Milli Q water* | *Sodium chloride* | *Milli Q water* |
|  | *Citric acid* | *Milli Q water* | *Milli Q water* |
|  | *Milli Q water* | *Milli Q water* | *MSG* |
|  | *Milli Q water* | *Milli Q water* | *Quinine* |
|  | *Mineral oil* | *Oleic acid* | *Mineral oil* |
|  | *Linoleic acid* | *Mineral oil* | *Mineral oil* |
